# Association of sarcopenia, pre-sarcopenia, and dynapenia with the onset and progression of locomotive syndrome in Japanese older adults: a cross-sectional study

**DOI:** 10.1186/s40101-023-00334-3

**Published:** 2023-08-03

**Authors:** Hungu Jung, Shigeharu Tanaka, Shusei Kataoka, Ryo Tanaka

**Affiliations:** 1 Graduate School of Humanities and Social Sciences, Hiroshima University, 1-7-1 Kagamiyama, Higashi-Hiroshima City, Hiroshima, Japan; 2 Department of Medicine for Integrated Approach to Social Inclusion, Graduate School of Biomedical and Health Sciences, Hiroshima University, 1-2-3 Kasumi, Minami-Ku, Hiroshima City, Hiroshima, Japan; 3 Department of Sports, Health and Well-Being, Faculty of Human Health Science, Hiroshima Bunka Gakuen University, 3-3-20 Heiseigahama, Saka-cho Aki-gun, Hiroshima, Japan; 4 Physical Therapy Major, School of Rehabilitation, Kanagawa University of Human Services, 1-10-1 Heiseicho, Kanagawa Yokosuka City, Japan; 5 School of Integrated Arts and Sciences, Hiroshima University, 1-7-1 Kagamiyama, Higashi-Hiroshima City, Hiroshima, Japan

**Keywords:** Locomotive syndrome, Muscle function, Muscle mass, Older adults, Sarcopenia

## Abstract

**Background:**

Sarcopenia commonly occurs in older adults with motor disorders requiring long-term care, and the clinical features of sarcopenia are associated with locomotive syndrome. Dynapenia is the age-related loss of muscle strength. However, the association of sarcopenia and dynapenia with the onset and progression of locomotive syndrome in older adults remains unknown. The current study aimed to determine the association of sarcopenia, pre-sarcopenia, and dynapenia with the onset and progression of locomotive syndrome in Japanese older adults.

**Methods:**

This study included older females (*n* = 264, 73.9 ± 5.8 years) and males (*n* = 92, 76.3 ± 6.1 years). Sarcopenia was defined as low muscle function and mass; pre-sarcopenia was defined as low muscle mass with normal muscle function; and dynapenia was defined as low muscle function without low muscle mass. Locomotive syndrome (stage 0–2) severity was determined using the stand-up test, the two-step test, and the 25-question geriatric locomotive function scale. Logistic regression analysis was performed to determine the relationship between sarcopenia category and locomotive syndrome stages.

**Results:**

Age (1.208, 95% confidence interval (CI) 1.124–1.298), sex (2.455, 95% CI 1.241–4.856), and BMI (1.211, 95% CI 1.077–1.361) were significant variables for determining locomotive syndrome stage ≥ 1, whereas pre-sarcopenia (0.543, 95% CI 0.331–0.891) and sarcopenia (1.664, 95% CI 1.005–2.755) were significant variables for determining locomotive syndrome stage 2.

**Conclusions:**

Only sarcopenia was associated with *locomotive syndrome* progression, while low muscle mass or low muscle function was not associated with locomotive syndrome. Gaining muscle mass accompanied by an increased muscle function for older adults is warranted to prevent locomotive syndrome progression in the super-aged society.

## Background

Locomotive function impairment is a cause of health disparities among older adults aged ≥ 65 years [1]. Older adults who experience difficulty walking, climbing stairs, going to the shop, or doing housework are at higher risk of requiring nursing services compared to those who do not experience the same difficulties [2]. In this context, the Japanese Orthopaedic Association (JOA) in 2007 proposed the concept of locomotive syndrome (LS) [3], which is associated with musculoskeletal disorders [1].

Aging has been associated with changes in body composition, such as skeletal muscle mass and body mass index (BMI). Accordingly, BMI has been associated with mortality, with individuals having an increased BMI with muscle mass loss being at a higher risk of death [4]. Sarcopenia and pre-sarcopenia are conditions characterized by changes in body composition and muscle function, particularly muscle mass loss, declined motor ability, or loss of muscle function, in older adults [5]. Previous reports have demonstrated that sarcopenia was common in older adults with motor disorders requiring long-term care [6] and that the clinical features of sarcopenia were subsequently associated with LS [7]. Gaining muscle mass in older adults may does not prevent muscle strength loss with aging, considering that muscle strength is lost at a considerably faster rate than muscle mass [8]. Clark and Manini [9] had proposed the term “dynapenia” to describe age-related loss of muscle strength against sarcopenia, which can be used in its original context to describe age-related loss of muscle mass. Muscle strength loss is an essential factor of LS [7]. To the best of our knowledge, the impact of muscle mass and/or function loss, including strength loss, in older adults with LS has yet to be investigated. Moreover, the association of sarcopenia, pre-sarcopenia, and dynapenia with the onset and progression of LS in older adults remains unclear. Confirming the extent of the association would certainly help prevent the onset and progression of LS due to musculoskeletal disorders in older adults.

Therefore, the current study aimed to determine the association of sarcopenia, pre-sarcopenia, and dynapenia with the onset and progression of LS in older adults. We hypothesized that muscle mass and/or function loss would be strongly linked to older adults with LS.

## Methods

### Study design

This cross-sectional study included Japanese community-dwelling older adults. All parameters were essential for assessing the subjects’ functional status and were not harmful. All procedures were conducted in accordance with the Ethical Principles for Medical Research Involving Human Subjects of 1975 stated in the World Medical Association Declaration of Helsinki. This research has been approved by the ﻿institutional review board of the authors’ affiliated institutions (approval number: 02–05).

### Setting

Participants were recruited from the staff of regional comprehensive support centers, community centers, and gymnasiums throughout Hiroshima Prefecture, Japan. A flyer with an outline of this study was also used for subject recruitment. Subject recruitment and data measurements were performed between November 2020 and December 2021.

### Participants

Community-dwelling individuals aged ≥ 65 years and individuals with independent mobility were included in this study. All participants provided written informed consent before participating. Whereas, individuals with suspected cognitive dysfunction and severe illness (unstable cardiovascular disease, stroke, serious breathing impairment, Parkinson’s disease, diabetic peripheral neuropathy, injury to the spinal cord, or rheumatoid arthritis) were excluded from this study to ensure the accuracy of responses to the questionnaire survey and prevent falls during measurement and worsening of diseases, respectively.

### Sarcopenia category

**Fig. 1:**
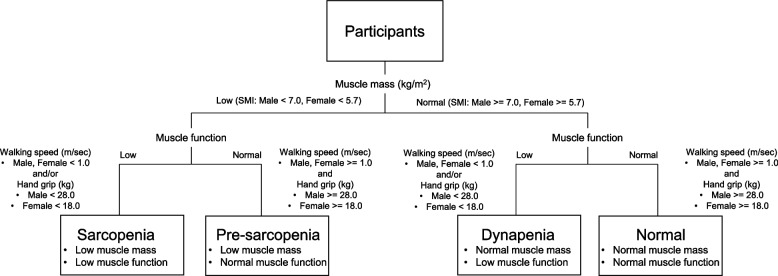
Methods of categorization based on skeletal mass index (SMI) and muscle function

Participants were classified into the normal, pre-sarcopenia, dynapenia, and sarcopenia groups according to a previous study [10] (Fig. 1). According to the diagnostic algorithm established by the Asian Working Group for Sarcopenia, sarcopenia was defined as the presence of both low muscle mass, as measured using the skeletal mass index (SMI), and low muscle function, as measured using hand grip and walking speed [11]. Pre-sarcopenia was defined as the presence of low muscle mass without low physical performance or low muscle strength based on the European Working Group on Sarcopenia in Older People (EWGSOP) definition [12]. Dynapenia was defined as the presence of low muscle function without low muscle mass [10].

### Skeletal muscle mass index

SMI, a skeletal muscle mass index, was measured through multifrequency bioelectrical impedance analysis (MF-BIA; InBody 270, Tokyo, Japan) [13]. InBody270 estimates SMI in absolute values (kg), which is subsequently calculated by height squared (m^2^). The technique also estimates body fat. The height was referred to as the most recent value of the subject. SMI measurements begin at 10 am or 2 pm for the convenience of participants. These time points may be 1–3 h after meals. The indoor temperature was set to keep a comfortable temperature by heating or cooling, depending on the season. The MF-BIA instrument is a common method used to measure body composition, such as SMI. Body mass index (BMI) was divided by the height in meters squared. This tool is instantaneous, versatile, convenient, and noninvasive for measuring bioelectrical impedance.

### Measurements of physical performances

Walking time is a reliable and valid method for measuring walking speed [14]. The test subjects walked along a 5-m path as instructed. A spare path of 1 m was provided for walking. The following instruction was provided for uniformity: “Walk forward at normal speed.” The subjects were only measured once.

Measuring hand-grip strength using a hand-held dynamometer has been reported to be reliable and valid [15]. In this study, a grip strength meter (TKK 5401 Grip-D; Takei, Niigata, Japan) was utilized for measuring grip strength. The test subjects held the grip strength meter with the pointer facing outward. The interphalangeal joints of the fingers were adjusted so that they were nearly at right angles prior to measurement. Measurements were recorded in kilograms. The average of each hand was used for subsequent analysis.

### Categorical outcome

The main outcome measure was LS severity, which was assessed using the LS risk test developed to detect motor dysfunction closely associated with disability. This test consists of the stand-up test, two-step test, and 25-question geriatric locomotive function scale, a self-administered questionnaire (GLFS-25) [16] proposed by the JOA. Previous studies have confirmed that this test had good validity, reliability, and feasibility [16, 17].

In the stand-up test, an individual who was able to stand from a 40-cm platform using both legs and stand from the same height using a single leg (both right and left side) was determined not to have LS (stage 0). An individual with LS stage 1 could not stand on a 40-cm platform with either leg, but could stand on a 20-cm platform with both legs. It was determined that an individual with LS stage 2 could not stand on a 20-cm platform with both legs but could stand on a 30-cm platform.

In the two-step test, the distance between the first standing line and the landing point’s toe was determined. This test was conducted twice, with the better result being recorded. The two-step test score was calculated by dividing the distance (cm) by the height (cm). Using this test, severity was categorized as follows: stage 0, 1.3 ≤ two-step test score; stage 1, 1.1 ≤ two-step test score < 1.3; and stage 2, two-step test score < 1.1.

Using GLFS-25, severity was categorized based on the following criteria: stage 0, 7 points < GLFS-25 score; stage 1, 7 points ≤ GLFS-25 score < 16 points; and stage 2, 16 points ≤ GLFS-25 score.

The final LS severity was determined after completing all three tests. Among the severity stages determined by the outcomes of three tests, the most severe stage was selected for further analysis.

### Statistical analysis

The Shapiro–Wilk test was used to verify the normality of data distribution, whereas Levene’s test was used to assess the homogeneity of variance. Independent sample t-tests were conducted when the data were homogeneous and normally distributed to compare variables between sexes, whereas Mann–Whitney *U* tests were applied whenever normality and homogeneity of variance were absent. Differences in the proportion of LS stages between sarcopenia categories were tested using chi-square tests. Analysis of variance (ANOVA) for background information was conducted separately for males and females to determine the subject characteristics across each sarcopenia category. The post-hoc test was performed using the Tukey method. Logistic regression analysis using forced entry method was performed to determine the relationship between sarcopenia category and LS. Sarcopenia category was entered into the regression model as independent variables. In logistic regression analysis, we used the deviation contrasts to compare the effect of each category (pre-sarcopenia, dynapenia, or sarcopenia) except the reference category (normal) to the overall effect. To adjust for the relationship between sarcopenia category and LS severity, age, sex, and BMI was included in the regression model as a confounding variable. In model 1, the dependent variable was the binary value of LS severity, which was either 0 or ≥ 1. The dependent variable in model 2 was a binary variable with an LS severity of ≤ 1 or 2. The significance level was set *p* < 0.05. SPSS (Version 18, SPSS, JAPAN) was used for all statistical analyses.

### Sample size

We followed standard methods for estimating the sample size using multiple logistic regression, with at least 10 outcomes needed for each included independent variable [18]. The number of variables to be fed into the independent variables of our logistic regression model was six (age, sex, BMI, pre-sarcopenia, dynapenia, and sarcopenia). Assuming an 20% prevalence of LS stage 0 based on a previous study showing an 19.0% prevalence of LS stage 0 (females, 18.7%; males, 19.6%) [19], 300 subjects (among whom 60 had LS stage 0) were required to appropriately perform logistic regression with six variables.

## Results

A total of 356 subjects participated in this study. The subjects’ background information is summarized in Tables 1 and 2. Females and males were significantly different in all variables (*p* < 0.05). ANOVA showed that all variables differed significantly according to sarcopenia category in both females and males. BMI in the dynapenia and normal groups in both females and males was significantly higher than in the pre-sarcopenia and sarcopenia groups (*p* < 0.05). The sarcopenia group was older than the pre-sarcopenia and normal groups in females while the sarcopenia group was older than the dynapenia group in males (*p* < 0.05).

**Table 1:** The background information of the subject (women)

Sarcopenia category	Overall	Sarcopenia	Pre-sarcopenia	Dynapenia	Normal	*P* value
Number	264	41	77	32	114	
Age, year	73.9	78.0	73.8	76.2	71.8	< 0.001
	(5.8)	(6.6)	(5.2)	(6.7)	(4.5)	P, N < S N < D
Height, cm	152.9	149.5	152.0	151.8	155.0	< 0.001
	(5.4)	(4.8)	(4.7)	(6.4)	(4.9)	S, P, D < N S < P
Weight, kg	52.9	46.9	48.2	56.0	57.3	< 0.001
	(8.0)	(6.4)	(5.0)	(8.2)	(6.9)	S, P < D, N
BMI, kg/m^2^	22.6	21.0	20.9	24.4	23.9	< 0.001
	(3.2)	(2.7)	(2.3)	(3.7)	(2.8)	S, P < D, N
SMI, kg/m^2^	5.8	5.2	5.3	6.0	6.2	< 0.001
	(0.6)	(0.3)	(0.2)	(0.3)	(0.4)	S, P < D, N
Walking speed, m/sec	1.3	1.0	1.4	1.0	1.3	< 0.001
	(0.3)	(0.3)	(0.2)	(0.3)	(0.2)	S, D < P, N
Hand grip, kg	21.0	17.5	21.4	16.7	23.7	< 0.001
	(4.3)	(2.8)	(2.1)	(5.3)	(3.6)	S, D < P < N
LS Stage 0, number	46	4	16	5	21	0.043^†^
	17.4%	9.8%	20.8%	15.6%	18.4%	
LS Stage 1, number	151	21	49	14	67	
	57.2%	51.2%	63.6%	43.8%	58.8%	
LS Stage 2, number	67	16	12	13	26	
	25.4%	39.0%	15.6%	40.6%	22.8%	

Upper row; average, lower row; (standard deviation)

*P* value (analysis of variance)

Data under the *P* values are the results of post-hoc test (Tukey)

^†^P-value (chi-square test)

*LS* locomotive syndrome, *S* sarcopenia, *P* pre-sarcopenia, *D* dynapenia, *N* normal

**Table 2:** The background information of the subject (men)

Sarcopenia category	Overall	Sarcopenia	Pre-sarcopenia	Dynapenia	Normal	*P* value
Number	92	17	19	17	39	
Age, year	76.3	82.7	78.1	75.1	73.0	< 0.001
	(6.1)	(4.9)	(6.8)	(5.4)	(3.6)	N < P, D < S
Height, cm	164.1	158.3	164.3	162.7	167.1	< 0.001
	(6.3)	(5.4)	(5.4)	(5.3)	(5.6)	D, S < N S < P
Weight, kg	63.0	55.4	57.4	67.3	67.3	< 0.001
	(9.1)	(6.7)	(6.3)	(6.3)	(8.8)	S, P < D, N
BMI, kg/m^2^	23.4	22.1	21.3	25.4	24.1	< 0.001
	(2.8)	(2.1)	(2.1)	(2.2)	(2.7)	S, P < D, N
SMI, kg/m^2^	7.1	6.4	6.5	7.5	7.6	< 0.001
	(0.7)	(0.5)	(0.3)	(0.5)	(0.5)	S, P < D, N
Walking speed, m/sec	1.2	1.1	1.2	0.9	1.3	< 0.001
	(0.2)	(0.3)	(0.2)	(0.1)	(0.2)	D, S < P, N
Hand grip, kg	33.1	25.7	31.5	35.3	36.1	< 0.001
	(6.3)	(5.3)	(3.2)	(6.9)	(4.6)	S < P < N S < D
LS Stage 0, number	20	2	3	3	12	0.006^†^
	21.7%	11.8%	15.8%	17.7%	30.8%	
LS Stage 1, number	47	4	12	9	22	
	51.1%	23.5%	63.2%	52.9%	56.4%	
LS Stage 2, number	25	11	4	5	5	
	27.2%	64.7%	21.0%	29.4%	12.8%	

Upper row; average, lower row; (standard deviation)

*P* value (analysis of variance)

Data under the *P* values are the results of post-hoc test (Tukey)

^†^*P* value (chi-square test)

*LS* locomotive syndrome, *S* sarcopenia, *P* pre-sarcopenia, *D* dynapenia, *N* normal

The results of logistic regression analysis are shown in Table 3. In model 1 (dependent variable; LS stage ≥  1), age (*p* < 0.001), sex (*p* = 0.010), and BMI (*p* = 0.001) were identified as significant independent variables. In model 2 (dependent variable; LS stage 2), the sarcopenia category (*p* = 0.026) was determined to be a significant independent variable, with the odds ratios for pre-sarcopenia (0.543, 95% confidence interval (CI) 0.331–0.891) and sarcopenia (1.664, 95% CI 1.005–2.755) being significant.

**Table 3:** Results of logistic regression analysis

Independent variables	Dependent variables	Model 1 (LS stage ≥ 1)				Model 2 (LS stage 2)			
		OR	95% CI		*P* value	OR	95% CI		*P* value
			Lower limit	Upper limit			Lower limit	Upper limit	
Age		1.208	1.124	1.298	< 0.001	1.098	1.048	1.151	0.001
Sex (1 = female)		2.455	1.241	4.856	0.010	1.268	0.708	2.272	0.425
BMI		1.211	1.077	1.361	0.001	1.040	0.946	1.142	0.418
Sarcopenia Category (Reference: Normal)					0.820				0.026
	Pre-sarcopenia	1.200	0.680	2.117	0.530	0.543	0.331	0.891	0.016
	Dynapenia	0.746	0.392	1.538	0.427	1.345	0.789	2.292	0.275
	Sarcopenia	1.222	0.567	2.635	0.610	1.664	1.005	2.755	0.048

*LS* locomotive syndrome, *OR* odd ratio, *CI* confidence interval

## Discussion

The current study investigated the association of sarcopenia, pre-sarcopenia, and dynapenia with the onset and progression of LS on a community of Japanese older adults. Our results revealed that in a logistic regression analysis using the results of the overall judgment with the three tests, the standard method to assess LS severity, age, sex, and BMI are significant variables for LS stage ≥ 1 as are pre-sarcopenia and sarcopenia for LS stage 2.

Musculoskeletal disorders were found to be associated with LS [7]. Sarcopenia characterized by muscle mass loss frequently occurred in older adults and was associated with a greater risk of incident disability [5, 6]. Recent studies have reported that sarcopenia can completely reflect serious health problems among older adults and that most older adults with sarcopenia had LS [19, 20]. LS with sarcopenia was found to be an essential factor in early identification of older adults who are at a higher risk of deterioration in health status and subsequent disability [21]. Considering the need for preventing disability of functional capacity, detailed evaluation of advancing LS stage is imperative. However, no study on community-dwelling older adults had examined the relationship between the classification of sarcopenia, pre-sarcopenia, and dynapenia by loss of muscle mass and/or function and the onset and progression of LS. Thus, this is the first study to report on the association of sarcopenia, pre-sarcopenia, and dynapenia with the onset and progression of LS.

BMI was a significant factor for the onset of LS, but it cannot be distinguished by the components of body weight, such as fat mass, muscle mass, bone, and organs. Sex was related to the onset of LS; i.e., females are more likely to have LS due to the difference in physical function between sexes, e.g., low muscle mass and weak muscle strength [22]. However, age was a significant factor for the onset of LS as females have more life extensity than males [23]. Therefore, we fully agree on the importance of preventing the onset of LS in super-aging societies, such as Japan.

The current study found that pre-sarcopenia was a significant variable of LS stage 2 with an OR of < 1, indicating that older adults with normal muscle function might not be diagnosed with LS stage 2. Individuals with pre-sarcopenia have normal muscle function evaluated using a 5-m walking speed and hand grip strength. Baseline walking speeds of ≥ 1.07 m/s in men and ≥ 1.02 m/s in women have been used for the clinical determination without a possibility for having a disability requiring long-term care within the Japanese population [24]. One study showed that grip strength was associated with mobility [25]. Specific grip strength thresholds have been used to identify older adults who likely have walking limitations, with walking speeds as slow as < 0.80 m/s [25]. Grip strength thresholds for older men (23.2–39.0 kg) and women (15.9–22.0 kg) have been reported [25]. In the current study, subjects with pre-sarcopenia showed a walking speed of 1.2 and 1.4 m/s and grip strength of 31.5 and 21.4 kg in older men and women, respectively, suggesting that keeping higher walking speed and normal grip strength could help prevent the progression of LS. Furthermore, our results showed that those with pre-sarcopenia displayed significantly lower weight and BMI than those in the normal and dynapenia groups, which can positively contribute to locomotion. EWGSOP defined pre-sarcopenia as a conceptual staging of sarcopenia representing the age-related decrease in muscle mass, indicating that pre-sarcopenia can develop into sarcopenia with weak low muscle function [12]. From this perspective, our results only indicate that despite a decrease in muscle mass, normal muscle function could help prevent the progression of LS.

Dynapenia was not a significant factor for LS stage 2, indicating that low muscle function alone may not be associated with LS progression, like low muscle mass alone did not contribute to LS stage 2. Dynapenia was a stronger predictor for falls, mortality, and activities of daily living in community-dwelling older adults [26–28]. Weak muscle strength, as one of low muscle function, compared with muscle mass, was more strongly associated with poor physical performance [29]. A longitudinal study reported that muscle strength decline in older adults is more rapid than the concomitant loss of muscle mass, indicating a reduction in muscle quality [30]. They also noted that maintenance or even muscle mass gain for the older adults did not necessarily prevent muscle strength loss [30]. This paradoxically emphasizes a need for muscle mass gain accompanied by increased muscle strength.

Muscle weakness can decrease physical function, diminish physical activity, and sometimes mobility disability [22, 29, 31], which is associated with low muscle mass [30]. Therefore, attention should also be given to older adults with dynapenia. Moreover, the hand grip strength test is more objective and safe, which indirectly evaluates locomotion ability [25].

In previous studies, the majority of older adults with sarcopenia were diagnosed with LS [19, 21]. In accordance with this, the current study added new evidence that sarcopenia was a significant factor for LS stage 2 but was unrelated to the onset of LS. The stand-up test, two-step test, and GLFS-25 assessing LS severity were highly correlated with lower extremity muscle strength, gait function, and activities of daily living, respectively [32]. In addition, our results revealed that low muscle mass and function were strongly associated with advanced LS severity, indicating that it is essential for increasing muscle mass and function in order to prevent the progression of LS.

However, older adults without sarcopenia, pre-sarcopenia, or dynapenia, were diagnosed with LS in our study: 93 females (LS stage 1, 67; LS stage 2, 26) and 27 males (LS stage 1, 22; LS stage 2, 5). This indicates that LS can occur or develop even in an older adult without losing muscle mass and/or function. A previous study reported that reduced static postural and balance ability assessed using a single-leg standing test was a predictive factor for the onset and progression of LS [33]. Therefore, other pathologies independent of low muscle mass and function should be investigated.

Our results have important practical implications for the risk of LS. Early detection of low muscle function is crucial for preventing the development of sarcopenia among older adults, consequently considerably reducing the likelihood of developing severe LS associated with functional disabilities and need for care service [34]. Furthermore, loss of muscle mass and function should be prioritized during evaluation. Therefore, exercise intervention programs for older adults should be recommended to gain muscle mass with an increase in muscle function, which would prevent the prevalence of sarcopenia and the progression of LS severity in older adults.

This study had several limitations. First, the results obtained from Japanese older adults cannot be generalized to all older adult populations given the differences in hand grip strength among races [35]. Second, given the cross-sectional nature of this study, the influence of sarcopenia, pre-sarcopenia, and dynapenia on LS over time may be difficult to elucidate within a specified period of physical ability decreasing by age. Further longitudinal studies are thus essential to evaluate the influence of sarcopenia, pre-sarcopenia, and dynapenia on LS over time. Third, this study defined dynapenia as low muscle function without low muscle mass; however, no internationally accepted definition of dynapenia has yet been established. Fourth, pre-sarcopenia was defined based on the EWGSOP in 2011 [12], which is not the current definition. Moreover, EWGSOP2 in 2019 did not argue about the definition of pre-sarcopenia, possibly aiming no merit in diagnosing pre-sarcopenia [36]. We demonstrated that an individual with pre-sarcopenia was not negatively associated with LS severity. Fifth, we used BIA methods to analyze body composition, and not dual-energy X-ray absorptiometry (DXA), which is the gold-standard technique. BIA methods strongly correlate with DXA methods, as reported in one study [37]. Thus, our results were considered reliable. Additionally, attention should be given to the data obtained using BIA methods because muscle mass is under or overestimated by BIA methods compared to DXA methods [37]. Sixth, we did not measure calf circumference to diagnose sarcopenia or pre-sarcopenia. Calf circumference is an anthropometric measurement to identify early a sign of sarcopenia and was strongly correlated with SMI [11, 38], thereby corroborating our findings. Finally, we did not standardize conditions to reduce the estimation error of participants’ body compositions: fasting or staying for 4 h with no food or drink consumption, absence of exercise in the previous day, absence of alcohol or diuretic drinks, normal hydration level, and a stable temperature of 23 ℃ [39, 40]. This is because of the characteristics of older adult participants. They may reject implementing standardized conditions for their health or daily habit. Thus, we set it up, considering their convenience and safety. The data in the current study may differ from those obtained under standardized conditions.

## Conclusions

Our results revealed age, sex, and BMI to be significantly associated with the onset of LS. Only sarcopenia was related to LS progression, while low muscle mass or low muscle function was not associated with LS. Therefore, gaining muscle mass accompanied by an increased muscle function for older adults is warranted to prevent LS progression in the super-aged society.

## Data Availability

Not applicable.

## Abbreviations

ANOVA: Analysis of variance
BMI: Body mass index
CI: Confidence interval
EWGSOP: European Working Group on Sarcopenia in Older People
GLFS-25: 25-Question geriatric locomotive function scale
JOA: Japanese Orthopaedic Association
LS: Locomotive syndrome
SMI: Skeletal mass index
